# Integrated cognitive and physical fitness training enhances attention abilities in older adults

**DOI:** 10.1038/s41514-022-00093-y

**Published:** 2022-08-30

**Authors:** Joaquin A. Anguera, Joshua J. Volponi, Alexander J. Simon, Courtney L. Gallen, Camarin E. Rolle, Roger Anguera-Singla, Erica A. Pitsch, Christian J. Thompson, Adam Gazzaley

**Affiliations:** 1 Neuroscape, University of California San Francisco, San Francisco, CA 94158 USA; 2 Department of Neurology, University of California San Francisco, San Francisco, CA 94158 USA; 3 Weill Institute for Neurosciences & Kavli Institute for Fundamental Neuroscience, University of California San Francisco, San Francisco, CA 94158 USA; 4 Department of Psychiatry, University of California San Francisco, San Francisco, CA 94158 USA; 5 Department of Physiology, University of California San Francisco, San Francisco, CA 94158 USA; 6 Department of Kinesiology, University of San Francisco, San Francisco, CA USA

**Keywords:** Cognitive ageing, Learning and memory, Neural ageing

## Abstract

Preserving attention abilities is of great concern to older adults who are motivated to maintain their quality of life. Both cognitive and physical fitness interventions have been utilized in intervention studies to assess maintenance and enhancement of attention abilities in seniors, and a coupling of these approaches is a compelling strategy to buttress both cognitive and physical health in a time- and resource-effective manner. With this perspective, we created a closed-loop, motion-capture video game (Body-Brain Trainer: BBT) that adapts a player’s cognitive and physical demands in an integrated approach, thus creating a personalized and cohesive experience across both domains. Older adults who engaged in two months of BBT improved on both physical fitness (measures of blood pressure and balance) and attention (behavioral and neural metrics of attention on a continuous performance task) outcome measures beyond that of an expectancy matched, active, placebo control group, with maintenance of improved attention performance evidenced 1 year later. Following training, the BBT group’s improvement on the attention outcome measure exceeded performance levels attained by an untrained group of 20-year olds, and showed age-equilibration of a neural signature of attention shown to decline with age: midline frontal theta power. These findings highlight the potential benefits of an integrated, cognitive-physical, closed-loop training platform as a powerful tool for both cognitive and physical enhancement in older adults.

## Introduction

The augmentation of deficient attention abilities is especially of interest for older adults, given well documented age-related declines in these abilities^1–9^, which are exacerbated in the presence of interference^2,10–13^. Over the last decade, there has been a surge of research aimed at improving cognitive abilities by harnessing neuroplasticity via cognitive training^13–17^. Cognitive training as a whole has come under warranted scrutiny given consistent shortcomings in study design and outcomes: (i) minimal extension of benefits beyond the training interventions themselves, (ii) lack of appropriate control groups, and (iii) absence of follow-up testing to assess sustainability of observed effects^18–20^. However, there have been several notable examples of cognitive interventions enhancing non-trained cognitive abilities in older adults^5^^,21–23^, with these findings aligning with systemic reviews supporting the utility of cognitive interventions in the older adult population^24–26^.

In contrast to the uncertainty surrounding the benefits of cognitive training, the utility of physical fitness interventions for older adults’ general health has been well established. Fitness-based interventions have also been shown to benefit cognitive control abilities^27–32^, with such findings hinting at the possibility that combining cognitive and physical training approaches may lead to greater cognitive benefits than either approach alone (although not supported to date^33–36^). The use of combined cognitive and physical interventions are especially compelling as a strategy to maintain both cognitive and physical health^37–39^, given that such a pairing can be a time- and resource-effective approach for addressing multiple risk factors in older adults. Indeed, combined training has been shown to result in greater participant enjoyment compared to either sequential training^40^ or physical exercise alone^41^. However, while the typical protocol of alternating between physical and cognitive training has shown to have some cognitive benefits^34,35,41–43^, this cumbersome tactic requires a multitude of human and material resources. Alternatively, one resource-effective approach in this arena has been the use of exergames; however, it remains unclear how effective such approaches are at improving cognitive and physical outcomes in aging populations^44–49^, leaving an open question of their overall utility.

**Fig. 1:**
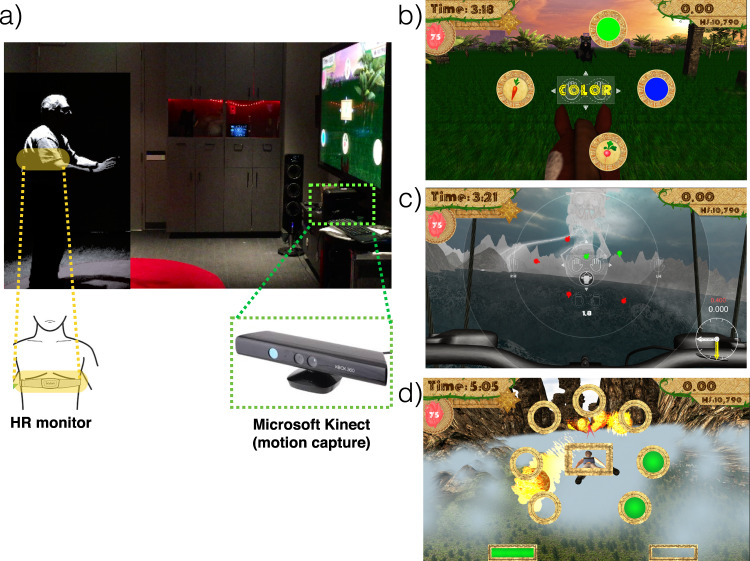
Body-Brain Trainer (BBT) platform. **a** Image of participant playing BBT. Highlighted is the use of a heart rate monitor to assess and adapt the physical intensity of gameplay in real-time, as well as the Microsoft Kinect™ motion-capture technology used to collect responses with one’s hands and/or feet based on the cognitive task presented on the monitor, and adapt the cognitive difficulty of each game in real-time. **b** Image of the task-switching module. **c** Image of the attention module. **d** Image of the working memory module.

We have previously demonstrated that cognitive interventions using closed-loop, adaptive algorithms^50^ designed to target specific cognitive abilities in older adults can improve task performance and neural measures of cognition^12,23,21,51,52^, with some evidence of these effects persisting well after the intervention period^21,53^. These interventions, delivered as engaging video games, have shown positive effects not only with older adults, but in diverse populations^54–57^, with previous work highlighting the possibility of returning performance on measures of cognitive control to young adult levels^5,21^. Here we created a novel exergame intervention for older adults (Body-Brain Trainer, BBT; Fig. 1) that requires participants to perform full-body, physical movements in response to cognitive challenges that engage different cognitive control domains^50,58^. As in our previous work^21,59,60^, we integrated real-time adaptivity using closed-loop mechanics for each cognitive ability being challenged. Given the movement demands of BBT, we also implemented an adaptive physical fitness challenge by using real-time heart rate data to titrate gameplay for a personalized and integrated training experience across both cognitive and physical domains (see Methods and Supplementary Materials for more details on the BBT software).

In the present study, we sought to evaluate the primary question of whether BBT could improve measures of attention and physical fitness in older adults beyond that of an expectancy matched, active placebo control group. To the best of our knowledge, this design is noteworthy given that the use of expectancy matching^61^ has been absent in all previous efforts examining cognitive benefits from combined approaches, including those studies designed to evidence potential synergistic effects through mechanistic control groups (see Supplementary Materials for a brief treatise describing our rationale for utilizing this particular control group). We also interrogated the following secondary questions: what is the neural mechanism underlying positive cognitive effects, do any observed cognitive improvements persist one year later without booster training sessions, does BBT result in older adults achieving comparable levels to young adults on our primary outcome measure, and does this intervention affect other measures of cognitive control (working memory).

## Results

### Participation and study overview

To interrogate the posed questions, we performed a double-blinded, randomized, placebo-controlled study where 49 healthy older adults (OA; mean age = 68.5 ± 6.3, 26 females) were randomly assigned to an intervention group (BBT: *n* = 24) or an active, expectancy-matched control group (Mind-Body Trainer (MBT): *n* = 25, see Supplementary Fig. 1 and Methods for details). A total of 41 of these participants (BBT = 21, MBT = 20) returned 1 year after completing their intervention to assess for the presence of sustained cognitive benefits (a CONSORT figure describing participant enrollment can be found at Supplementary Fig. 2). Details regarding improvements on the BBT training intervention itself can also be found in the Methods, as well as Supplementary Figs. 3–6).

### Sustained attention

**Fig. 2:**
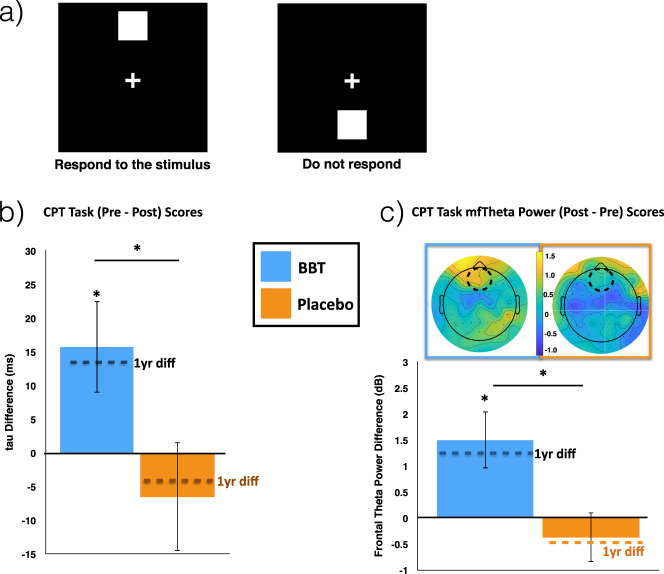
CPT task. **a** Stimuli and protocol for the attention without distraction (CPT) task. **b** Bar graphs illustrating the group mean change in ex-gaussian tau (pre - post, with + values demonstrating improvement in tau over time) for each group, with the dashed line (1-year diff = pre - 1-year) illustrating the change in tau at the 1-year mark. **c** Bar graphs and topographic plots illustrating the group mean change in midline frontal theta power (post - pre) for each group, with the dashed line on the bar graphs illustrating the change in power at the 1-year mark (1-year - pre). The dashed circle on the topographic plot illustrates the electrodes where statistical analyses took place. *p < 0.05. Error bars represent s.e.m.

**Fig. 3:**
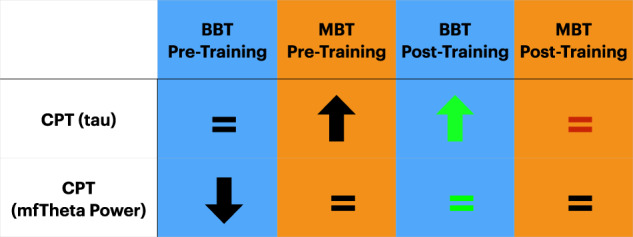
Comparison of BBT and MBT performance versus younger adults. **⇒** performance significantly lesser than that of young adults. **⇒** performance significantly better than that of young adults. **⇒** performance equivalent to that of young adults. Green = Significant improvement from pre-training. Red = Significant decline from pre-training.

Here we used a vigilance task as our primary cognitive outcome measure to assess the intervention’s impact on attention abilities. This task was a customized Continuous Performance Task (CPT), which is a modified version of a well-validated sustained attention task, the Test of Variables of Attention (TOVA)^62^, used as an outcome measure in our previous intervention studies^21,23,57^ (Fig. 2a). Using a repeated measures ANOVA to evaluate change in CPT performance from pre- to post-intervention (see ‘Statistical methods’ in Methods), we found a significant time (Pre, Post) by intervention (BBT, MBT) interaction (*F*_*1,45*_ = 7.49, *p* = 0.009, Cohen’s *d* = 0.79), with performance improvements (i.e. a reduction in tau from pre to post) in the BBT group (Δ = + 15.73 msec improvement, paired *t*-test: *t*_*23*_ = 2.29, *p* = 0.03), but not in the MBT group (Δ = −6.46 msec decline, paired *t*-test: *t*_*24*_ = −1.60, *p* = 0.13; Fig. 2b, and Table 1 for values). When this measure was compared to a separate untrained cohort of young adults (*n* = 51; Fig. 3) who completed this task in a single visit, the BBT group did not show expected age-related performance differences at baseline; i.e., the BBT group exhibited equivalent performance to young adults (independent *t*-test: *t*_73_ = 0.42, *p* = 0.67; see Table 1 for values). However, following training, the BBT group showed significantly lower tau (i.e., better performance) than young adults (independent *T*-test: *t*_74_ = 2.20, *p* = 0.03). Finally, in evaluating stability of performance gains over time, follow-up tests 1 year later revealed that neither group showed significant differences from post-training performance levels (paired *t*-tests: BBT: *t*_20_ = 1.61, *p* = 0.12; MBT: *t*_19_ = 0.34 *p* = 0.74; see Table 1).

**Table 1:** Group means and standard deviations for each primary measure at each timepoint for each group.

	BBT	MBT	Young Adult
Primary Measures			
cPT (tau) Assessment (msec)			
Pre-Training	72.5 (39.7)	52.2 (31.2)	68.1 (29.4)
Post-Training	55.8 (19.0)^a^	58.7 (39.4)	
1-Year	59.1 (17.4)	57.9 (30.4)	
Secondary Measures			
Working Memory Task, No Distractor Condition^b^ (tau)			
Pre-Training	29.7 (14.1)	24.1 (10.0)	(n/a)
Post-Training	26.0 (12.0)	24.2 (12.0)	
Exploratory Measures			
Frontal Theta Power during CPT (dB)			
Pre-Training	0.77 (1.85)	1.67 (2.27)	2.11 (1.43)
Post-Training	2.28 (2.68)^a^	1.30 (2.20)	
1-Year	1.80 (2.25)	1.05 (1.11)	
Filter (RTV) Assessment, Set Size 3 (msec)			
Pre-Training	544.1 (229.3)	470.8 (138.9)	409.5 (162.5)
Post-Training	412.6 (148.9)^a^	459.2 (107.2)	
1-Year	487.0 (297.7)	378.2 (123.0)^c^	
Frontal Theta ITC during Filter (PLV)			
Pre-Training	0.24 (0.09)	0.28 (0.09)	0.25 (0.06)
Post-Training	0.28 (0.11)^†^	0.26 (0.09)	
1-Year	0.23 (0.05)^c^	0.21 (0.08)^c^	
Diastolic Blood Pressure (mmHg)			
Pre-Training	78.72 (5.25)	76.23 (5.91)	(n/a)
Post-Training	73.00 (5.68)^a^	77.52 (6.32)	
Limit of Stability Assessment (balance; m/s)			
Pre-Training	3.88 (1.34)	4.29 (1.26)	(n/a)
Post-Training	4.59 (1.26)^a^	4.30 (0.99)	
Basic Response Time (mean RT; msec)			
Pre-Training	409.1 (83.3)	372.4 (63.3)	(n/a)
Post-Training	390.7 (74.2)	373.6 (79.0)	

No significant difference between groups observed at baseline (see Statistics for details).

^a^Post-training performance significantly different than Pre-training performance (^†^*p* = 0.08).

^b^Performance on No Distractor condition shown, with similar results observed (not reported) on the Attend Distractor and Ignore Distractor conditions.

^c^Post-training performance significantly different than 1-Year performance.

We also performed a second analysis of attention (not part of the ‘parent’ clinicaltrials.gov registration assessments, thus considered to be exploratory) to assess how a more challenging complex visual discrimination task with varying levels of distraction would be impacted by these interventions (see Supplementary Fig. 7a and Methods for task details). The same pattern of results as described for CPT in terms of both the statistical interaction and follow-up tests 1 year later were observed on the complex visual discrimination task (see Supplemental Materials and Supplementary Fig. 7b).

### Neural correlates of sustained attention

In addition to measuring cognitive performance, participants also underwent electroencephalography (EEG) recordings during the CPT task to examine the neural metric of midline frontal theta power, given that it has been associated with sustained attention abilities^63,64^ and is a sensitive marker of changes in attention abilities following interventions^21,53,54^. A repeated measures ANOVA revealed a significant group by time interaction (*F*_(1,43)_ = 6.61, *p* = 0.014, Cohen’s *d* = 0.74; Fig. 2c), with post-hoc paired-samples *t*-tests showing a significant increase in power following the intervention for the BBT group (paired *t*-test: *t*_*23*_ = −2.77, *p* = 0.011), but not in the control group (paired *t*-test: *t*_*20*_ = 0.79, *p* = 0.44). Once again, the same pattern of results was also observed for the complex visual discrimination task using comparable EEG analyses (see Supplemental Materials and Supplementary Fig. 7c).

When compared to the group of young adults, the older adult BBT group showed expected age-related reductions in pre-training theta power (independent *T*-test: *t*_56_ = 3.10, *p* = 0.003). After the intervention period, the BBT group’s post-training power increased to a level equivalent to that of young adults (independent *T*-test: *t*_57_ = 0.11, *p* = 0.91, see Fig. 3, and Table 1 for values). With respect to assessing the stability of these changes 1-year later, neither group showed significant differences from their post-training levels (paired *t*-tests: BBT: *t*_18_ = 1.16, *p* = 0.26; MBT: *t*_14_ = 0.95, *p* = 0.36; see Table 1 for values).

### Fitness measures

**Fig. 4:**
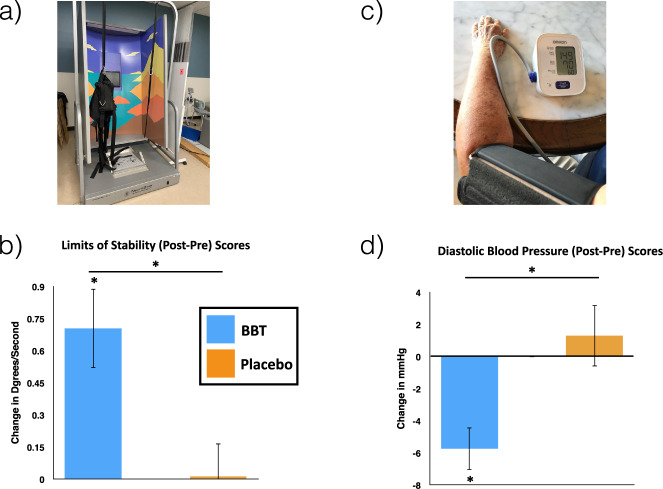
Improvements in Physical Outcomes Assessments. **a** Limits of Stability (LoS) assessment on the NeuroCom balance manager system^150,151^. The LoS is a center-out postural stability test that serves as a basic balance quantification metric. During this task, increases in movement velocity have been associated with decreases in fall risk in older adults^152^. **b** There was a significant group x time interaction in movement velocity of the LoS task (repeated measures ANOVA: *F*_1,40_ = 7.814, *p* = 0.008, Cohen’s *d* = 0.90). A paired *t*-test analysis revealed that the BBT group showed a significant increase from pre to post (*t*_18_ = 4.53, *p* < 0.001), whereas the control participants showed no difference (*t*_22_ = −1.02, *p* = 0.32). Note there was no difference at baseline between groups (independent *t*-test: *t*_43_ = 1.77, *p* = 0.08.) **c** Diastolic blood pressure assessment. Diastolic blood pressure is a predictor of overall physical health and has been found to be decreased through exercise interventions^153,154^. **d** There was a significant group x time interaction (repeated measures ANOVA: *F*_1,40_ = 8.43, *p* = 0.006, Cohen’s *d* = 0.78). A paired *t*-test analysis revealed that the BBT group showed a significant decrease from pre to post (*t*_19_ = 4.31, *p* < 0.001), whereas the control participants showed no significant change following their intervention (*t*_21_ = 0.46, *p* = 0.65). Note there was no difference at baseline between groups (independent *t*-test: *t*_44_ = 1.05, *p* = 0.30). Error bars represent s.e.m.

We assessed whether the BBT intervention led to measurable improvements in physical fitness measures beyond that of the control group by focusing on metrics of balance^65–69^ and blood pressure^70–73^, given their association with real-world health outcomes in older adults. Using a repeated measures ANOVA to test for differential group improvement over time, we observed a significant group by time interaction on a balance measure (i.e., the limits of stability test associated with risk of falling;^74,75^ *F*_(1,44)_ = 8.37, *p* = 0.006, Cohen’s *d* = 0.90, Fig. 4a); notably, the BBT group significantly improved on this stability measure (paired *t*-test: *t*_21_ = −3.81, *p* = 0.001) whereas the MBT group did not (paired *t*-test: *t*_23_ = −0.81, *p* = 0.94). With respect to blood pressure, a repeated measures ANOVA revealed a significant group by time interaction of diastolic blood pressure (*F*_(1,41)_ = 9.32, *p* = 0.004, Cohen’s *d* = 0.78, Fig. 4b), with the BBT group significantly decreasing their diastolic blood pressure after training (paired *t*-test: *t*_21_ = 4.35, *p* = 0.001) while the MBT group did not (paired *t*-test: *t*_20_ = 0.68, *p* = 0.51). See Methods and Supplementary Materials for details on systolic blood pressure as well as other exploratory physical fitness metrics.

### Working memory and basic response time assessments

We also explored whether BBT led to an improvement on a working memory task (described in the trial registration as a secondary outcome) that has been shown to improve in previous intervention studies of older adults^11,21,55,76^ (see Methods for task details). A repeated measures ANOVA of tau using a within-subject factor of Session (Pre, Post) and a between-group factor of Study Group (BBT, MBT) did not demonstrate a Study Group x Time interaction for any of the three working memory conditions (no distractions, ignore distractions, attend to distractors), indicating that tau on this working memory test did not differentially change between study groups (*F*_(1,39)_ ≦ 0.95, *p* ≧ 0.34, see Table 1 for no distractor condition values). Similarly, independent *t*-test analyses showed that there was no group difference in performance post-training on any of the three conditions (*t*_(42)_ ≦ 1.46, *p* ≧ 0.52).

Finally, we administered a Basic Response Time (BRT) task as a measure of simple motoric response time to ensure that any differences observed between groups were not due to improvements in basic motoric speed (see Methods for task details). A repeated measures ANOVA with a within-subject factor of Session (Pre, Post) and a between-group factor of Study Group (BBT, Control) did not reveal an interaction, indicating that RT did not differentially change as a function of training between study groups (*F*_(1,40)_ = 2.16, *p* = 0.15, see Table 1 for values). Similarly, there was no RT performance difference post-training between the BBT and MBT groups (independent *t*-test: *t*_(42)_ = 0.44, *p* = 0.67). This same analysis approach revealed the same outcome for RTV, with no observed interaction (repeated measures ANOVA: *F*_(1,40)_ = 0.31, *p* = 0.58) or difference post-training (independent *t*-test: *t*_(42)_ = 1.5, *p* = 0.13). These results suggest that differences found on the primary outcome measures cannot be attributed to improvements in basic motoric speed or motoric variability.

## Discussion

### Overview

Here we demonstrate that an integrated cognitive-physical fitness intervention (BBT) improved measures of physical fitness, as well as objective measures of attention beyond an expectancy-matched, active placebo control group. After the intervention, their post-training cognitive performance reached comparable, and in one case superior, levels to younger adults, with these gains being maintained 1 year later. Here we discuss the implications of these findings and how they relate to other efforts aimed at improving cognitive function in older adults.

### Physical, cognitive, and neural enhancements

Given the movement demands of BBT, it was unsurprising yet reassuring that engagement led to improved balance, consistent with results from other exergaming studies in older adults^77,78^. However, this is the first study of its kind that also evidenced diastolic blood pressure improvements beyond a control group, suggesting that the adaptive fitness mechanics spurred a physiological benefit not previously seen before. BBT also had a positive effect on attention abilities in older adults, in alignment with other combined cognitive-physical intervention studies that evidenced enhanced attention in older adults^33,35,42^. However, the benefits observed here were attained with a significantly shorter training regimen (8 weeks) compared to the majority of these other combined studies (typically between 12–16 weeks, with some lasting up to 30 weeks), and with much less logistical burden by the training in each domain being integrated rather than split across different training days. Another key difference from previous studies was the incorporation of adaptivity, not just for cognitive challenges but also for physical fitness demands, which allowed us to titrate the physical training appropriately to each individual’s fitness level. We hypothesize that the integration of the cognitive and physical training within a video game experience may have spurred the observed benefits after a relatively abbreviated intervention time.

The attention-based improvements reported here are comparable to those found in other aging intervention studies from our group that utilized different closed-loop adaptive mechanics to improve attention both with^23^ and without distraction^21^. Attention improvements documented using the same CPT task have also been observed in a recent meditation-based intervention study in younger adults^57^. This convergence of attention improvements emerging from different types of closed-loop interventions in older adults and other populations is particularly compelling^54,55,79,80^. Indeed, the results from these studies suggest the potential for individuals to select their preferred digital treatments without concerns about differential efficacy.

While there is ample evidence that combined interventions in older adults can have positive effects on overall cognitive function^45,81–83^, few studies have included neuroimaging assessments to examine the underlying mechanisms of improvements observed^36,84,85^. Studies involving such combined approaches have reported increased cerebral glucose metabolism in frontal and sensorimotor regions^36^, enhanced fMRI resting state functional connectivity between frontal and temporal regions^85^, and increased fMRI resting state synchronization at temporal and cerebellar regions^84^. These findings highlight distinct neural metrics involving ‘task-free’ measures across different brain regions associated with improved cognitive performance, as brain-behavioral correlations were observed in each study. Here, we observed that the activation of a frontal top-down network accompanied improved attentional performance without external distraction, as well as increased trial-by-trial neural consistency that corresponded with improved attentional focus when distractions were present. These findings are aligned with our previous work in older adults evidencing similar top-down theta power enhancements following a closed-loop video game intervention (NeuroRacer)^21^, as well as improved theta coherence following a distraction training intervention in both older humans and rats^23^. Thus, empirical support exists for the idea that midline frontal theta is an especially sensitive marker of attentional control changes in intervention studies^53^.

### Comparisons to young adults and long-term maintenance of benefits

The present findings offer a mixed interpretation of whether the observed attention-based improvements were a remediation of age-related deficiencies or the intervention enhanced non-deficient processes. The older adult BBT cohort exhibited age-related deficits in midline frontal theta power that increased to levels equivalent to young adults after training, unlike the control group. However, the BBT cohort showed comparable performance levels at baseline (CPT tau) to the young adults, although after training they were significantly better than their younger counterparts. Previous studies have evidenced age-related declines on these same metrics^21,86^. The current results support a compensation effect^87,88^, given that improvements in the BBT group led to performance levels exceeding that of younger adults and suggest that integrated cognitive and physical approaches designed to augment plasticity in neural systems may have the potential to remediate certain aging deficits^23,89^.

Other work has suggested that combined interventions may have a particular advantage with respect to long-term maintenance of observed benefits^83^. We offer evidence here demonstrating that each group had comparable performance levels at the 1-year mark compared to levels attained immediately post-training. These findings should be considered with caution given that very few intervention studies targeting cognitive control abilities have evidenced persisting effects well past the initial treatment period^90–97^. Indeed, our own work failed to realize the persistence of the more distant transfer effects several years later^53^, and even here the sensitivity of these measures one year later is unclear. While it has been argued that the value of these types of interventions are dubious without the demonstration of sustained benefits beyond the initial treatment period^98,99^, we posit that these findings better highlight the potential utility of incorporating additional booster sessions for sustaining benefits.

### Conclusions

Here we observed that BBT led to improvement in the ability of healthy older adults to stabilize their attention from moment to moment on a vigilance task beyond that of an expectancy-matched, placebo control group. Beyond the methodological approach used here, these results are highly relevant for older adults given the steady advancement of innovative approaches designed to help these individuals with their cognition and physical fitness. The present findings support and extend those studies that reported positive effects of combined interventions on cognitive function^45,83,100,101^, with accompanying physical benefits supporting the use of such technology to realize meaningful benefit in both cognitive and physical domains. Thus, comparable combined cognitive and physical interventions may be a viable time- and resource-effective approach for older adults to maintain (or even enhance) both their cognitive and physical health.

### Limitations

There are several limitations with the present study that are important to note. First, this work does not directly interrogate whether BBT leads to beneficial effects beyond those achieved with training cognitive or physical abilities in isolation. Several studies and meta-analyses have already interrogated this question^33–36^, with these works demonstrating that a combined approach does not always yield synergistic effects beyond cognitive training alone. Furthermore, due to the experimental design, we cannot directly assess how much of the observed attention improvements can be attributed to the individual physical and cognitive training components incorporated in BBT. Indeed, both cognitive interventions and physical fitness training have been shown to induce attention improvements and positive functional changes in the prefrontal cortex (i.e. increased task-based activations)^96,102,103^ in line with present findings.

Second, while we observed improvements on distinct measures of attention, as in previous work, improvements did not emerge on a working memory outcome measure as it did in previous intervention-based studies^21,55^ (see Supplementary Materials). One possible explanation for this result is that BBT disproportionately engaged attention-based circuitry during training, given that both working memory^104^ and cognitive flexibility abilities^105,106^ call upon attentional resources for their successful utilization. Thus, attention abilities were engaged during each BBT module across the entire intervention periods. This interpretation, while speculative, is consistent with previous work that targeted cognitive flexibility and observed improvements on the same outcome measures used here (CPT and the working memory task), as well as on the same neural measures^21^. Third, while expectations regarding potential improvements from the interventions for each group were comparable, our experimental design cannot directly address the influence that distinct training experiences might have had on the results (e.g. researcher supervision, location of treatment, warm-up periods, etc.). Nevertheless, it is important to restate that the goal here was to compare our exergame intervention to an expectancy-matched, placebo control. The present findings support the conclusion that these effects were not driven by expectations and encourage future mechanistic work to better understand the contributions of each aspect of the combined training experience. Finally, all of the conclusions associated with the 1-year mark analyses should be interpreted with caution as our interpretations are based on non-significant differences. This analytic approach was motivated by the relatively small sample size present at the follow-up point, as well as the desire to provide a perspective on the potential persistence of such effects.

## Methods

### Participants

The study was approved by the Committee on Human Research at the University of California, San Francisco, and retrospectively registered on ISRCTN registry [ISRCTN [66423499] to distinguish the goals and approach taken here from those of the ‘parent’ registration (clinicaltrials.gov submission NCT03032796). This study was designed to gauge the feasibility and potential efficacy of using the BBT intervention compared to an expectancy matched active placebo group, for a subsequent mechanistic trial exploring synergistic effects of a combined intervention versus individual components alone (as described in the ‘parent’ clinicaltrials.gov submission). For continuity and cohesion, we used the same primary (CPT) and secondary (Working Memory Task) outcome measures listed in the parent clinicaltrials.gov registration. Furthermore, here we also examined a subset of the exploratory outcomes listed in the parent registration (blood pressure, event-related spectral perturbation derived from EEG) as well as two new exploratory measures (Filter Task, Limit of Stability Assessment). 49 healthy, older adult (OA) individuals consented to participate in this study. OAs were randomly assigned (block size of 5) to a training group (body-brain trainer; BBT; *N* = 24, mean age = 68.8 ± 5.9, 13 females) or an active, expectancy-matched control group (mind-body trainer; MBT; *N* = 25, mean age = 68.20 ± 6.75, 13 females). All participants were from the San Francisco Bay Area and recruited through online and newspaper advertisements. All participants had normal or corrected-to-normal vision, had no history of stroke, traumatic brain injury, or psychiatric illness, were not taking psychotropic, hormonal, or cardiovascular medications, and did not have any physical or mental conditions that may interfere with their daily activities (e.g., migraine headaches, substance abuse, neuropathy. Similar to our previous work, all participants reported playing less than 2-hours of video games per month, and completed a general health questionnaire reviewed by the study team assessing each individual’s current state of physical fitness to ensure that they could safely engage with the physical aspects of the BBT platform. All participants gave written informed consent, and were paid $15/hour for their in-lab and at-home participation. All participants were encouraged to not change any aspects of their daily routine (e.g. to change exercise habits) for the duration of the study.

### Neuropsychological battery

Prior to experimental testing, all participants were evaluated on 3 measures probing for cognitive impairments and depression (the Montreal Cognitive Assessment (MoCA^107^; minimum score of 26; the Geriatric Depression Scale (GDS)^108^; the PHQ-9^109^) as well as nine neuropsychological tests. These nine tests were subdivided into related domains and composite scores of each were calculated for each of the following:

1. Immediate Memory—consisted of the five immediate recall trials from the California Verbal Learning Test (CVLT-II)^110^.
2. Delayed Memory—the long delay free and cued recall trials as well as the Recognition measure from the CVLT-II.
3. Processing Speed—Digit Symbol task, Executive Composite-DKEFS Trails Condition 2 (numbers only)^111^.
4. Cognitive Flexibility (Task Switching)—DKEFS Trails Condition 4 (number-letter switch).
5. Fluency—Verbal Fluency (Animals^112^, D- Words (MoCA).

Color vision deficiency was assessed with Ishihara’s Tests for Colour Deficiency^113^. To be included in the study, all individuals were required to be within 2 standard deviations (SD) of age-matched controls on all five of the composite scores. Participants were also excluded if two or more composite scores exceeded 1.5 SDs. This procedure provided a thorough characterization of the cognitive status of each OA participant in multiple domains while simultaneously ensuring that their cognitive faculties were comparable to that of their age-matched peers. All participants tested within two SDs of the normative values established for each of these measures.

### Study design

For those participants randomized to the BBT group, they were asked to come to Neuroscape at UCSF 3 days per week, 1 hour per visit (36 min of on-task training time per session, 24 minutes allocated for warm up/cool down/breaks) for 8 weeks (24 hours total, 14.4 hours of actual training time). Each of these visits were accompanied by an onsite trainer to facilitate the training experience for the participant and ensure that training was being completed in a safe manner. For those participants randomized to the MBT group, they were loaned an iPad tablet (9.7 inch screen size; 1024 × 768 screen resolution) for their training session following their ‘Pre-training’ assessment, and were instructed to train with their assigned task at home for six weeks, 5 days per week, with 30-minute training sessions per day, for a maximum of thirty 30-minute training sessions (15 hours of training total). All MBT participants were instructed to train sitting down with the tablet on a flat surface, such as a table, in a location with minimal external distraction.

We used a study design that involved several layers of blinding: (i) data was collected by a group of research associates who were blinded to the identity of the intervention group, (ii) data analysis (which was anonymized to conceal intervention group identity) was performed by a different group of researchers, and (iii) participants were blinded to the group assignment following randomization procedure, which was done prior to the first participant visit to the lab. Blinding began at the point of recruitment, where all participants were informed that they were being recruited for a study designed to test the efficacy of software interventions for improving cognition in a variety of domains. Neither the BBT nor the MBT participants were aware of the other group or the task that they trained with. Both groups were administered the same instructions and brief overview of the goals of the study, namely to determine if the training game could improve cognitive abilities. Thus, all participants were told that they were part of an active intervention to improve their cognitive abilities (see below for details on how we established matched expectancy^61^). Finally, one study coordinator was informed of the treatment assignments, as their role was solely to provide technical and other support during the training.

All participants reported to our UCSF Neuroscape laboratories prior to training (‘Pre-training’ session) and following the completion (‘Post-training’ session) of training (1-week grace period from start/end of training) to compete a battery of cognitive and physical outcome measures to assess training-related changes. Participants were invited to return to the laboratory 1 year after their post-training outcome assessment to evidence which, if any, positive training effects persisted in the cognitive domain. Over the course of the study, 3 participants in the BBT group and 2 participants in the MBT group voluntarily withdrew from the study, resulting in the complete pre- and post-training datasets from 24 BBT participants and 25 MBT participants. One additional participant had a non-training-related adverse event which caused them to withdraw from the study prior to being randomized to a group. We were unable to collect EEG data from three participants at pre-training due to equipment malfunctions, and, due to technical issues with photodiodes, we were unable to time-lock the EEG to the event onsets for several other participants (*n* = 4 at pre-training, *n* = 4 at post-training). There was one participant who did not return for their post physical assessment.

### Intervention descriptions

#### BBT paradigm

BBT is comprised of three modules, with each targeting a different aspect of cognitive control: visual search tasks for attention (with increasing distraction), spatial span/multiple object tracking tasks for working memory, and a task-switching paradigm targeting goal-management/cognitive flexibility abilities. There are also three different tasks with ascending difficulty within each module, such that advancing to the next level engages a fresh challenge while maintaining interest (for example, a change from a spatial span condition to a multiple object tracking condition with working memory demands). Comparable to our previous work using cognitive measures alone^21,59,60^, here we integrate real-time adaptivity for both the cognitive and physical aspects of the gameplay. For each cognitive task, difficulty scales on a trial-by-trial basis, with a correct trial performed within a thresholding-determined response window leading to shorter response window by 10 msec, and an incorrect trial leading to a lengthening of the response window by 30msec (thus, a 1”up”/3”down” staircase). These cognitive adaptive algorithms are designed to assure participants remain at an ~80% rate of accuracy, a level that is not too easy nor too hard, so that it is enjoyable and engaging. For the physical training, difficulty is tied to the demands associated with the distance an individual must travel for a given response and the amount of time allocated to complete this response. These movement-related aspects are directly responsive to whether heart rate is below/within/above a predetermined heart rate window to ensure a moderately intense workout that does not impede the ability to perform the cognitive task. For example, if one is playing the game *below* their assigned heart rate range, the software will automatically increase the distance that the participant must move to respond with their hands/feet on each trial until their heart rate is within the specified range. Training sessions are linked, such that the next session begins at the level attained at the end of the previous session. Participants are provided two types of feedback: (1) real-time feedback—indicating whether the participant successfully detected or classified the target and (2) punctuated feedback—participants advance through a series of “levels” that are reported at the beginning and end of each run.

### BBT module descriptions

(i) BBT Attention. This module demands an active scan of the screen in search of a target, much like traditional visual search tasks^114^. This module involves a constantly evolving amount of cued information as well as number of incongruent distracting elements, such that participants experience less cued information while experiencing more and more distracting elements as they advance. Participants are required to quickly identify the direction of a probe target that is facing at a right angle (up, down, left, right), and are aided by the presence of directional cue indicating in which location of the screen the target will appear amongst distracting elements. Responses are made by reaching their hands to indicate the direction of the probe, with the additional physical challenge of running in place if the target is up or down. Prior to each level, participants completed a thresholding session to determine the optimal starting point from both a cognitive and physical perspective. After completing their initial 7 training sessions, participants advance to LEVEL 2 of this module which entailed facing a greater challenge: here they encountered an increase in the number and salience of distracting elements, including the presence of congruent distractors, as based on their performance on the previous trial. After completing 14 training sessions, participants moved on to LEVEL 3 of this module: here participants performed the same task as before, but now without the aid of a directional cue. Participants only receive game points when they correctly perform a given trial faster than the predetermined, personalized threshold determined at the beginning of each level to optimize the attentional engagement.

(ii) BBT Working Memory. This module engages spatial working memory resources similar to the Corsi block task^115–117^, requiring individuals to memorize an additional stimulus following two consecutive correct responses, with two consecutive incorrect trials leading to one element being subtracted. Participants memorize the location of objects on screen followed by a 5–7 second delay period during which the participants perform a directed physical movement, with a correct response leading to a greater number of potential targets to be memorized on the next trial (and vice versa). Responses are made with both hands and feet by reaching/kicking targets, with additional physical challenges (making a woodchopping motion) occurring during the delay period. Prior to each level, participants completed a thresholding session to determine the optimal starting point from both a cognitive and physical perspective. After completing 7 training sessions, participants are asked to also memorize and report the sequential order in which the targets originally appeared on the screen (LEVEL 2), thus increasing the spatial working memory load. After 14 training sessions, participants perform a working memory/multiple object tracking task that requires memorizing and tracking the targets as they become invisible and move amongst a sea of moving objects (LEVEL 3). Participants receive game points when they correctly complete a working memory trial faster than a predetermined, personalized threshold so as to challenge the underlying cognitive working memory circuitry.

(iii) BBT Task Switching. This module challenges cognitive flexibility resources by requiring participants to rapidly switch their focus based on distinct rules, much like a traditional task-switching paradigm^118–120^. Here a morphing algorithm is used to titrate the perceptual similarity of the target presented, such that a correct trial makes a subsequent exemplar morph more similar to the probe presented (and vice versa). Participants are presented with exemplar objects along with a target, and move their hands to the target object that is most similar to the exemplar presented. For example, when a greenish-blue target appears, participants decide whether the image is more GREEN or more BLUE. The target changes its degree of likeness to each exemplar following each trial, with each correct response morphing the probe towards an indistinguishable 50/50 ratio of each exemplar (and vice versa). Prior to each level, participants completed a thresholding session to determine the optimal starting point from both a cognitive and physical perspective. After completing 7 training sessions, the presented probes now have features that integrate two rule bases (LEVEL 2, e.g. both Color and Shape, so a BLUE square), creating greater cognitive demands, similar to interference generated by a Stroop task. Finally, after 14 training sessions, participants perform the same task, but the exemplars now spawn in random locations across the screen, heightening the cognitive demands further by requiring visual search (LEVEL 3). Participants receive game points when they perform a trial as fast or faster than a predetermined, personalized threshold to pressure underlying goal-management circuitry.

### BBT cognitive and physical training calibration

For each cognitive task, difficulty was initially determined through a pre-training assessment to determine an optimal training threshold, then scaled on a trial-by-trial basis throughout the training experience. The associated physical challenge for each cognitive task was driven via motion capture of one’s hands and feet, where the distance that an individual had to reach/kick to respond during the cognitive task was directly tied to a participant’s predetermined optimal training heart rate. For example, if an individual’s heart rate was below their training window, then the distance an individual had to traverse to respond on a given trial increased.

This continuous cognitive scaling occurred in parallel with an adaptive cardiovascular challenge, with a pre-training VO_2_ max assessment determining an optimal training window with respect to one’s heart rate using indirect calorimetry^121^ to calculate a more precise level of cardiovascular fitness for each individual. More specifically, this involved participants completing a treadmill graded exercise VO_2_ Max test to subsequently determine an individual’s maximal heart rate, which subsequently was used to determine the heart rate percentiles (60–70% of one’s max HR, 70–80% of one’s max HR, etc.) at which participants would train at during the BBT training experience. This predetermined heart rate window was used as a baseline comparator against one’s current heart rate, with the responses needed for a given cognitive task scaled to be closer or further away than a previous trial without impeding the ability to perform the cognitive task.

### MBT battery

To mitigate any potential placebo effects brought on by participant expectations, we identified a suitable active control condition based on participant predictions of potential training-related gains as in our previous work^57^. We identified a set of three commercially available iOS apps (Supplementary Fig. 1) that were matched to our BBT program in terms of expectation of improvement on our cognitive outcome measures (see below for description of the statistical selection process). Those apps were a language learning app (Duolingo; www.duolingo.com), a Tai Chi app (Tai Chi Step by Step; www.imoblife.net), and a logic games app (100 Logic Games; www.andreasabbatini.com/LogicGames.aspx). For Duolingo, participants were given a choice of which language they wanted to learn from those available on the app. Within the app, we set a 10 min training time per day. During training, the app takes users through a series of modules that increase in difficulty and are only unlocked sequentially following completion of an earlier module. Modules are organized topically (e.g., Food, Animals, Phrases, etc.) and each module contains listening, speaking, vocabulary, and translation tasks and culminates with a topic quiz. At the end of each lesson the app provides a progress report showing learning “streaks” and the accumulation of “lingots” (Duolingo currency). These feedback features are meant to keep participants motivated. For Tai Chi, users simply open the app and select from a series of modules that provide detailed and easy-to-follow instructions on how to perform many basic, intermediate, and advanced Tai Chi movements and is geared toward beginners with no Tai Chi experience. Each description can be read or listened to and is accompanied by an animation. Users were instructed to then practice the exercise themselves several times after each lesson. The logic games app is comprised of a series of “puzzle sets” that revolve around a particular theme and which get progressively more difficult as people advance. The puzzles are similar to the more well-known Sudoku puzzles, but provide a more engaging experience with colorful icons, unique rule sets for each theme, and increasing difficulty. For each puzzle, users are given a task (e.g., plant trees according to specific rules), a time limit, and several hints that they can unlock. Participants were instructed to spend approximately 10 min with each app each training day (5 days per week for 6 weeks). For Duolingo, the time was set internally in the app. For the other two apps, participants self-timed their training, and they recorded their time on a training log. All participants completed the MBT intervention at home using an iPad Mini 2 (iOS version 8.2; Apple) that was supplied by Neuroscape. On the day they were given their iPad to begin training, participants were also provided an instructional binder with instructions on how to play, a calendar for recording notes and comments throughout their training experience, and were given e-mail support throughout the intervention period. Throughout the intervention period, technical support for the iPads and all software was provided via e-mail, phone, and in-person contact, when needed.

Because these apps were commercial apps and not designed to send data to our server, we took additional steps to track compliance in the MBT group. On the day they were given their iPad to begin training, participants were also provided an instructional binder with instructions on how to play, a calendar for recording notes and comments throughout their training experience, and were given e-mail support throughout the treatment period. They were instructed to try to split their time equally among the three apps. Upon completion of the training, researchers confirmed the participants’ logged training times.

### Behavioral cognitive control assessments

#### Attention without distraction

A custom continuous performance task (CPT) designed in Presentation (http://neurobs.com) was used to assess vigilance without distraction. This tool is a modified version of a well-validated vigilance task (Fig. 2a), the Test of Variables of Attention (TOVA), that we have used as an outcome measure in previous intervention studies. As our metric of interest, we focused on the ex-gaussian tau of response times, a non-parametric measure of distribution skewing that quantifies attentional lapses by examining the distribution of long response times^122–125^. Several studies have demonstrated a clear advantage of this measure over traditional measures of response time (although we report on traditional metrics of response time and response time variance in the Supplemental Materials) given that it explains a greater proportion of variance without needing to employ data trimming techniques^126–128^. Importantly, this measure has been shown to reflect performance inconsistencies that are especially present in both healthy older adults and those with mild cognitive impairment^129^, and has been used to support neural noise^130^ and dedifferentiation^131–133^ theories of cognitive aging.

For the present study, we adapted the task for use with EEG recordings, which requires many trials with an actual response. In this task, participants maintain fixation on a central crosshairs and gray squares are shown on a black background at the top or bottom of the field of view. Stimuli are presented frequently at the top of the screen as a 4:1 ratio of targets to nontargets and participants are instructed to only respond to these stimuli. Participants completed 2 blocks of 125 trials with 100 targets per block, yielding 200 total targets and 50 non-target trials.

#### Working memory fidelity task

We used a delayed recognition working memory paradigm designed to measure changes in participants’ ability to maintain an accurate mental representation of items in working memory either in presence or absence of distracting or interfering information. We have used versions of this task in numerous previous studies^11,21,55,76^. To summarize briefly, this paradigm consisted of three different conditions that were presented in blocks: (1) no distraction (ND), (2) Ignore distractor (ID, distractor was present, but participants were informed that the distractor was to be ignored), and (3) Attend Distractor (AD, participants were required to make a judgment about the interfering stimulus). Each run was preceded by an instruction slide informing the participant which condition they would be performing. Each trial began with the presentation of a face displayed for 800 ms, followed by a delay period (3 s), the presentation of a face stimulus as a distractor in the ID and AD conditions (800 ms), a second delay period (3 s), and the presentation of a face probe (1 s). The participants were instructed to make a match/nonmatch button press response at the probe as quickly as possible, without sacrificing accuracy. This was followed by a self-paced intertrial interval (ITI). The experiment was programmed in E-Prime (https://pstnet.com/products/e-prime/) and the stimuli were presented on a CRT monitor.

#### Basic response time task

Here we administered a basic response time (BRT) task as a measure of simple motoric response time to ensure that any differences observed between groups were not due to differences in basic motoric quickness. Basic motor speed was assessed in the form of a simple target-detection task during their pre-training assessment, to ensure that any training-related enhancements in performance were not attributed to a general motoric speed increase, but rather to enhancements in cognitive control processes. On this task participants pressed a keyboard button as quickly as possible upon the appearance of a circle at the center of the screen (50 trials). Average response time (the time between the target appearance and button press) was assayed as a unit of basic motor speed^134^.

#### Complex visual discrimination amid distractions

For this exploratory measure of attention in the presence of variable distraction (considered exploratory given that it was not described in the parent clinicaltrial.gov submission), we used a custom Filter Task^135^ (Supplemental Fig. 7a) to assess how well participants were able to identify targets in the presence of task-irrelevant information^57,136,137^. The experiment was programmed in MATLAB’s psychophysics toolbox (http://psychtoolbox.org/) and the stimuli were presented on a CRT monitor. In this task, participants must attend to an array of different numbers of items (either 1 or 3 red rectangles) with or without the presence of 2 visual distractors (2 blue or green rectangles): set size 1 no distractors (1 total item), set size 1 with distractors (3 total items), set size 3 no distractors (3 total items), set size 3 with distractors (5 total items). Half of each of the trials for each condition began with a cue indicating the participant should attend to either the left or the right side of the screen. The procedure for each trial began with a 750 ms fixation cross following by a right/left cue (200 ms) and then a 300 ms blank ISI. Next, a sample set from one of the four conditions was shown for 200 ms followed by a 900 ms blank delay and then a probe set containing the same number of red rectangles as in the sample in either the same orientation or with a single rectangle of altered orientation (50% of each). The probe screen remained visible until participants responded with a “Yes” or “No” button press indicating whether or not one of the attended rectangles changed orientation. Participants completed 8 blocks of 80 trials, yielding 160 trials per condition. Participants were instructed to respond as fast as possible without sacrificing accuracy. As in our previous work^57^, we focused on Response Time Variability (RTV) as our measure of interest here.

### Neural cognitive control assessments

#### EEG recordings

Neurophysiological data were recorded during each cognitive control assessment using an active two head cap (Cortech Solutions) with a BioSemiActiveTwo 64-channel EEG acquisition system in conjunction with BioSemiActiView software (Cortech Solutions). Signals were amplified and digitized at 1024 Hz with a 16-bit resolution. Anti-aliasing filters were used and data were band-pass filtered between 0.01–100 Hz during data acquisition. For each EEG recording session, a 1 × 1-inch white box was flashed for 10 ms at one of the corners on the stimulus presentation monitor at the start of each trial. A photodiode (http://www.gtec.at/Products/Hardware-and-Accessories/g.TRIGbox-Specs-Features) captured this change in luminance to facilitate precise time-locking of the neural activity associated with each sign event. During the experiment, these corners were covered with tape to prevent participants from being distracted by the flashing light.

#### EEG preprocessing

Preprocessing was conducted using the EEGLAB software^138^. Noisy channels were identified upon initial visual inspection, were removed from the data, and interpolated using a spherical spline interpolation, using the average signal of the surrounding channels to reconstruct the data in the removed channel. The data were then down-sampled to 1024 Hz to reduce the computational demand without losing any important information in the data. A finite impulse response filter with a high-pass cutoff of 1 Hz was applied to remove drift, and then a low-pass filter at 40 Hz was applied to remove high frequency noise. Ocular correction was performed by using ICA to isolate and remove activity induced by eye-blinks and lateral eye movements from the signal. The data were then re-referenced to the average signal of all channels. Epochs of −1000 ms to +1000 ms were generated for each stimulus type for subsequent analyses. Epochs containing excessive peak-to-peak deflections (±100 µV) were removed.

#### Midline frontal theta analyses

Midline frontal theta (4-7 Hz) power (mft power) has been implicated in sustained attention abilities^21,139–141^, including correlating with RTV across the lifespan^142^, that can evidence the engagement of the prefrontal cognitive control processes during a given task. This particular marker has also demonstrated a level of sensitivity in revealing changes following a digital intervention in multiple studies^21,23,53,54,57^. Time series were created by resolving 4–40 Hz activity using a fast Fourier transform (FFT) in EEGLAB in epochs from −1000 to +1000 msec. Midline frontal theta total power analyses (evoked power + induced power) across trials from the vigilance task was conducted by resolving 4–40 Hz activity using a fast Fourier transform in EEGLAB, with these values subsequently referenced to a −900 to −700 pre-stimulus baseline (thus relative power (dB)). After mft relative power was computed, we specifically interrogated a predetermined cluster of frontal electrodes (Fz, FPz, AF3, AF4, and AFz) at the time window of maximum power, as this is the same approach that we have previously utilized in several intervention studies interrogating mft power changes^21,53,54,57^.

### Physical outcome measures

To capture changes in physical fitness due to training during the study, all participants underwent a comprehensive physical outcomes assessment in addition to the cognitive assessments described above. Our targeted outcome measures were a senior-specific measure of stability indicative of fall risk^74,75^ as well as a physiological measure of fitness and health (diastolic blood pressure)^72^. Our decision to focus on diastolic (rather than systolic) blood pressure was based on diastolic blood pressure being a proportionally larger contributor to mean arterial pressure (MAP)^143^, with MAP being an important predictor of cardiovascular disease^144–146^. Descriptions of each exploratory fitness measure evaluated, including systolic blood pressure, are described in the Supplemental Materials; note that these measures came from the Senior Fitness Test (SFT) to assess global physical performance^147^.

### Training-related expectancy ratings

In an effort to identify an expectancy matched placebo versus the BBT intervention, we first compared expectancy on 10 different apps to BBT in a sample of 261 participants (Female = 135, mean age = 33.5). Each participant was randomly assigned to view a video of BBT or one of 10 potential apps (*n* = 15–30 individuals per app) which we did not hypothesize would improve cognitive abilities. They were then asked to rate the extent to which the presented intervention would lead to improvements on these outcomes, using a Likert scale measuring 1 (no improvement) to 7 (a lot of improvement). Following this first round of testing, we selected the three apps that had the highest expectancy matching scores, and subsequently collected new expectancy data in a sample of 76 young adults to assess the level of expectancy involving the combination of these three apps (*n* = 37) versus BBT (*n* = 39). Here we found no differences in participant expectations of improvement on our CPT task (*t*_74_ = 0.24, *P* = 0.62, 95% CI: −1.3 to 0.77) or on our filter task (*t*_74_ = 0.55, *P* = 0.16, 95% CI: − 0.30 to 1.8), suggesting that we had comparable expectancy on this battery of control apps as compared to the BBT intervention with respect to our cognitive control outcome measures.

To determine if these expectancy findings held with older adults, we had 91 older adults complete this same expectancy survey. These older adults were shown a recording of either the BBT (*n* = 46, mean age = 64.4, Female = 28) or the MBT (*n* = 45, mean age = 63.2, Female = 31) interventions and subsequently shown a video describing our cognitive outcome measures. We observed no significant difference between interventions in terms of participant expectations of improvement on the CPT (independent *t*-test: *t*_89_ = −1.84, *p* = 0.069, with the MBT intervention showing a trend towards having greater expectation of improving on this measure than the BBT group) and the Filter tasks (independent *t*-test: *t*_89_ = −0.682, *p* = 0.50). To ensure that these findings extended to the present study, we asked all BBT and MBT participants to complete a comparable version of this survey after they had completed their first few days of training on their assigned intervention to see if their experience led to different expectancy-based perceptions. This was motivated especially by the fact that the MBT training experience was distinct from the BBT training experience in that they completed their training at home on a tablet by themselves, whereas the BBT group trained at a laboratory on a large screen TV in the presence of a trainer. Once again, we observed no significant difference between interventions in terms of participant expectations of improvement on the CPT (independent *t*-test: *t*_36_ = 1.26, *p* = 0.21, MBT mean = 5.0, BBT mean = 5.4) and the Filter tasks (independent *t*-test: *t*_36_ = 0.64, *p* = 0.53, MBT mean = 5.0, BBT mean = 5.3).

### Statistical analysis approach

Analyses were conducted by researchers who were blind to group membership. To test for training effects on each of the collected outcome measures, we used an repeated measures ANOVA approach as in our previous work^21,23^. Statistically, no between-group differences assessed using independent *t*-tests were observed for at baseline involving: (1) age (*t*_47_ = 0.35, *p* = 0.73); (2) gender (*t*_47_ = −0.15, *p* = 0.88); (3) CPT performance (*t*_45_ = 1.84, *p* = 0.07); (4) Filter task performance (*t*_46_ = 1.34, *p* = 0.19); (5) mft power (*t*_44_ = 1.70, *p* = 0.10); (6) mft ITC (*t*_41_ = 1.11, *p* = 0.28); (7) limits of stability (*t*_44_ = 1.05, *p* = 0.30); (8) diastolic blood pressure (*t*_43_ = 1.77, *p* = 0.08). For those measures that showed a trend towards a group difference at baseline (CPT performance, mft power, and blood pressure), we performed an analysis of covariance (ANCOVA) with post-training performance as the dependent variable, pre-training performance as the covariate, and group as the fixed factor. Our reasoning for using this approach is that this analysis accounts for variation around the post-test means that arises from the variation where participants began at pre-test^148^. Using this approach, we observed a nearly significant group difference following training for the CPT task (*F*_(1,46_ = 3.85, *p* = 0.056), a significant group effect for mft power (*F*_(1,44_ = 4.35, *p* = 0.043) and a sigshynificant group effect for diastolic blood pressure (*F*_(1,42_ = 6.93, *p* = 0.012), supporting the assertion that the reported ANOVA effects were not driven by trending baseline differences between groups. ﻿For post-hoc analysis of the within-group changes, we performed two-tailed, paired-sample *t*-tests on each group separately to test for significant differences between each testing session. We report Cohen’s *d* for all significant (*p* < 0.05) and trending (*p* < 0.10) ANOVA results, using the Hedges and Olkin correction^149^ for small sample bias.

## Supplementary information

Supplemental Materials

## Data Availability

The datasets generated during and/or analyzed during the current study are available from the corresponding author upon reasonable request.
