# Supplementary material for: Integrated cognitive and physical fitness training enhances attention abilities in older adults
Source: NPJ Aging. 2022 Aug 30;8(1):12. doi: 10.1038/s41514-022-00093-y (PMC9427998; doi:10.1038/s41514-022-00093-y)
Supplement: Supplementary file 1 — Supplemental Materials [file 41514_2022_93_MOESM1_ESM.pdf]

Supplementary Materials

Distinctions between different control groups for cognitive intervention studies

The field of cognitive intervention studies is still in its infancy, but it has been developing at a rapid pace. A key aspect of the experimental design of these studies that has come under scrutiny (justifiably) is the choice of control group against which an intervention is compared. There have been several seminal articles written on this topic in recent years[1,2], including one by our group focused specifically on closed-loop cognitive interventions[3]. We and the others argue that an important distinction must be made between two broad types of control groups: no-contact controls and active controls. Until recently, the vast majority of cognitive intervention studies employed a no-contact control group. These passive control groups are useful for understanding how outcome measures are influenced by practice effects stemming from performing repeat assessments, but an active control group advances the interpretability of a study because it controls for both practice effects and other non-specific placebo effects.

Active control groups can further be divided into placebo controls or mechanistic controls. A placebo control group involves a participant cohort that engages in another intervention that matches participants' expectations of positive outcomes, but which does not contain any of the "active ingredients" hypothesized to drive the effectiveness of the intervention. Note that we performed an independent study on our designed intervention (BBT) and the active control group (MBT) to document that the *expectations of improvements* on our outcome measures were matched across each cohort. This design is meant to parallel the use of placebo controls that are routinely used in pharmaceutical intervention studies, allowing one to correctly surmise that any improvements by the intervention group beyond the control group were not driven by expectancy-based placebo effects at the outset of a trial. In contrast, a mechanistic control is an active control that is exactly matched to the main study intervention with only a single factor varied.

An example of this can be found in our previous work involving a cognitive training intervention for older adults ("NeuroRacer"; Anguera et al., 2013)[4]. In that study, the NeuroRacer training group trained on a multitasking condition ('respond to signs and drive a car') exclusively during the intervention, whereas the mechanistic control group divided their time between a 'respond to signs only' and a 'drive only' training experience. Thus, this mechanistic control was matched for all factors except the presence of interference arising from the multitasking experience. As such, this design, while being more challenging to find significant differences given the similarity of the training experience between the study groups, does allow more extensive assessment of the *mechanism of action* of a successful treatment by isolating the active ingredient of the effects.

Emerging from this debate about what constitutes an appropriate control condition is the perspective that the initial trial of a novel intervention should be one that employs an expectancy-matched, placebo control group[1,2]. Boot and colleagues (2013) described the importance of this approach as follows:

"*Without measuring and controlling for placebo effects, such studies provide little more than speculation about the causes of improvements…Researchers, reviewers, and editors should no longer accept inadequate control conditions, and causal claims should be rejected unless a study demonstrably eliminates differential placebo effects.*"

This is the choice that we made for this study, as this first step is critical before undertaking a much larger, much more resource-intensive mechanistic study. More specifically, without the demonstration of significant effects against a placebo control, we feel that undertaking a larger mechanistic study would be premature given the scale and resources necessary. Thus, the findings of a study involving an expectancy-matched placebo control group can provide a template for future work involving both mechanistic control groups to more fully explore the underlying mechanism(s) of action.

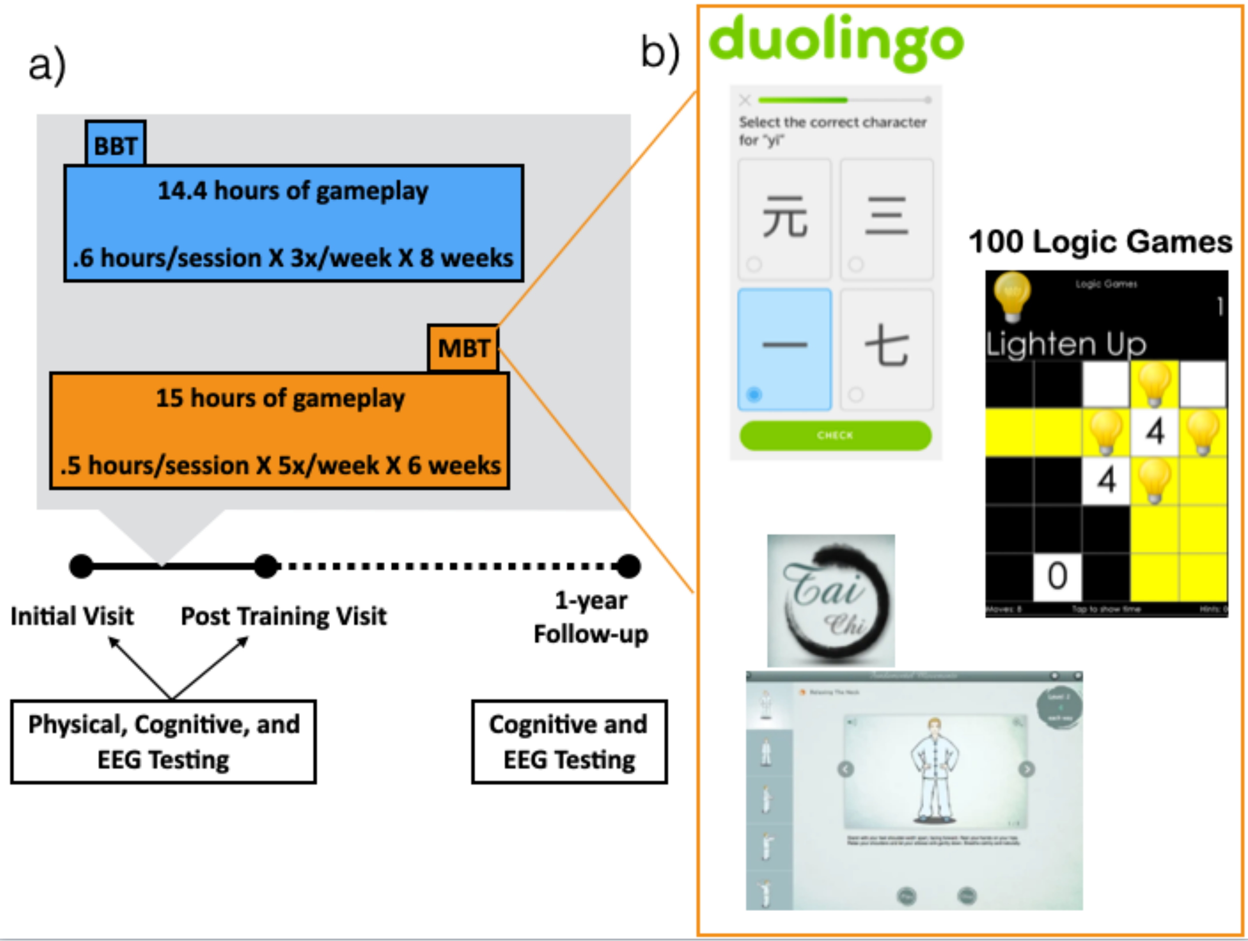

<u>Supplemental Figure 1. Trial Design and Mind-Body Trainer modules.</u> a. An overview of the study timeline as well as the breakdown in gameplay for Body-Brain Trainer (BBT) and Mind-Body Trainer (MBT), the active control. b. We identified a set of three expectancy matched apps to be used as an active control group for BBT: duolingo™, 100 Logic Games™, and Tai Chi™. Participants played each game for ten minutes a day, five times a week, for six weeks.

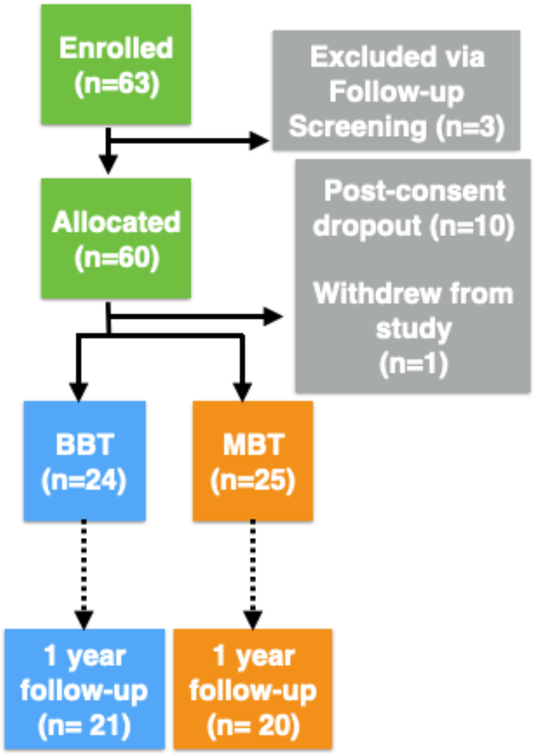

Supplementary Figure 2. CONSORT diagram. Enrolled= how many individuals passed neuropsychological assessment. Excluded via follow-up screening = When confirming an individual wanted to participate, these (3) individuals stated that they could not or no longer wanted to participate. Allocated: Individuals who were consented and confirmed participation interest. Post-consent dropout = after consenting to be in the study, these (10) individuals decided to drop out of the study. 5 individuals dropped out during the preassessment tasks, while the other 5 dropped out during training. Withdrew from study = had a non-study related adverse event precluding continued participation. BBT = Body-Brain Training group, MBT = Mind-Body Training group.

BBT training improvements:

Here we describe the BBT training improvement on the BBT platform itself as a quality control check to demonstrate that participants improved over time on both the cognitive and physical aspects associated with this intervention. With respect to BBT training itself, we found that participants in the BBT group demonstrated increased performance across their training sessions as measured by the amount of points accrued (paired t-test: $t_{21} = 9.40$, $p< 0.001$, **Supplementary Figure 3**), with these points accumulating as a function of training at or above their predetermined cognitive and physiological baseline thresholds (see **Methods** for details). Looking deeper into the individual components contributing to these points, we observed improved performance accuracy (paired t-test: $t_{21} = -3.34$, $p = 0.003$, **Supplemental Figure 4**) as well as increased heart rate (paired t-test: $t_{21} = -2.31$, $p = 0.031$, **Supplemental Figure 5**) over the course of training, suggesting that, unsurprisingly, cognitive and physiological performance on the intervention itself improved over time. These findings begged the question of whether those individuals who put the most effort into their physical fitness training, in terms of having an elevated heart rate, also showed the greatest improvement in performance accuracy across their training modules over time. This was indeed the case regardless of the stage of the intervention, as we observed a significant correlation between accuracy and heart rate averaged over the initial two days of training ($r_{22} = 0.49$, $p = 0.02$; **Supplemental Figure 6a**) as well as over the final two days of training ($r_{22} = 0.70$, $p = 0.0001$; **Supplemental Figure 6b**).

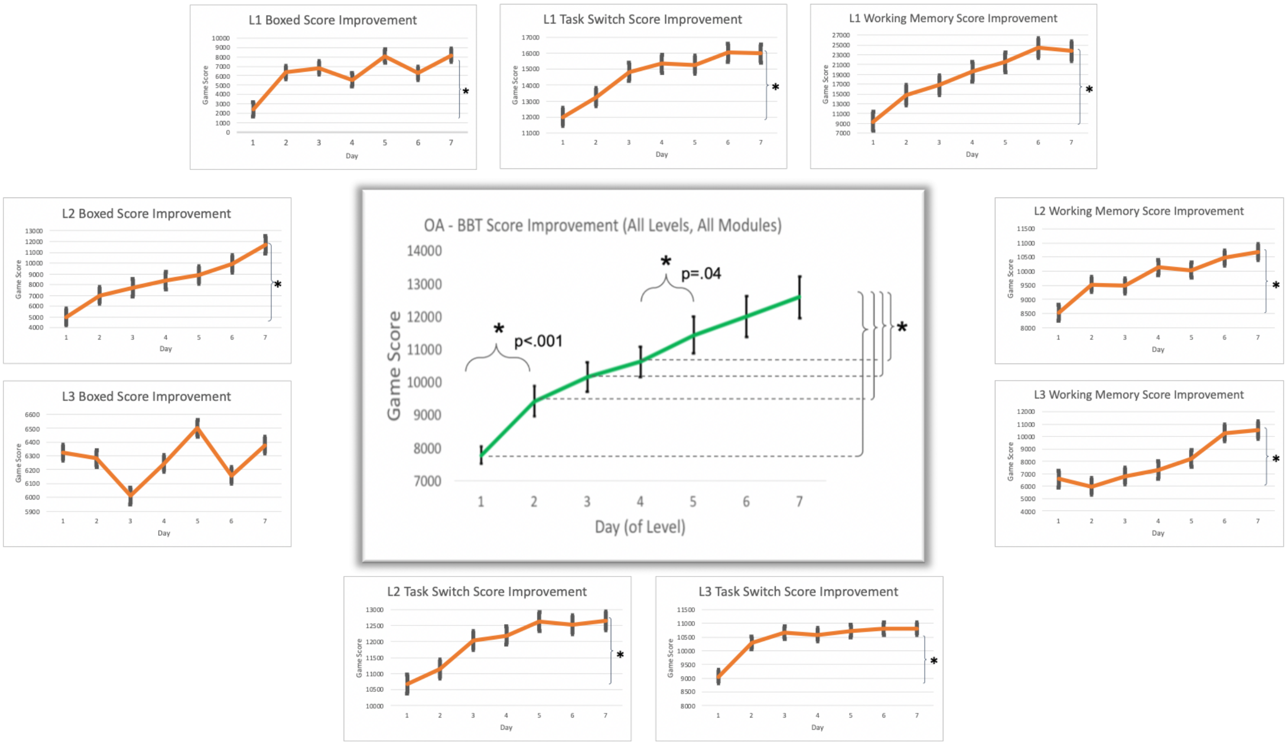

Supplemental Figure 3: Intra-intervention training improvement

During training on BBT, participants significantly increased their game score from the start to the end of each module for 8 out of 9 modules. Note: game score is dependent on correct answers, difficulty of task, time to reach the correct target, and the distance of those targets to the center of the screen. Taking the average of the nine BBT modules (center figure), participants significantly increased their game score from the start of training (Days 1 and 2, mean= 8640.63 points, s.e. = 492.25) to the end (Days 6 and 7, mean= 12297.48 points, s.e. = 739.50, Δ = 3656.86 points, paired t-test: t_{21} = -9.396, p = < 0.001, 95% CI: 2847.52 to 4466.19). * = p< 0.05 Error bars represent s.e.m.

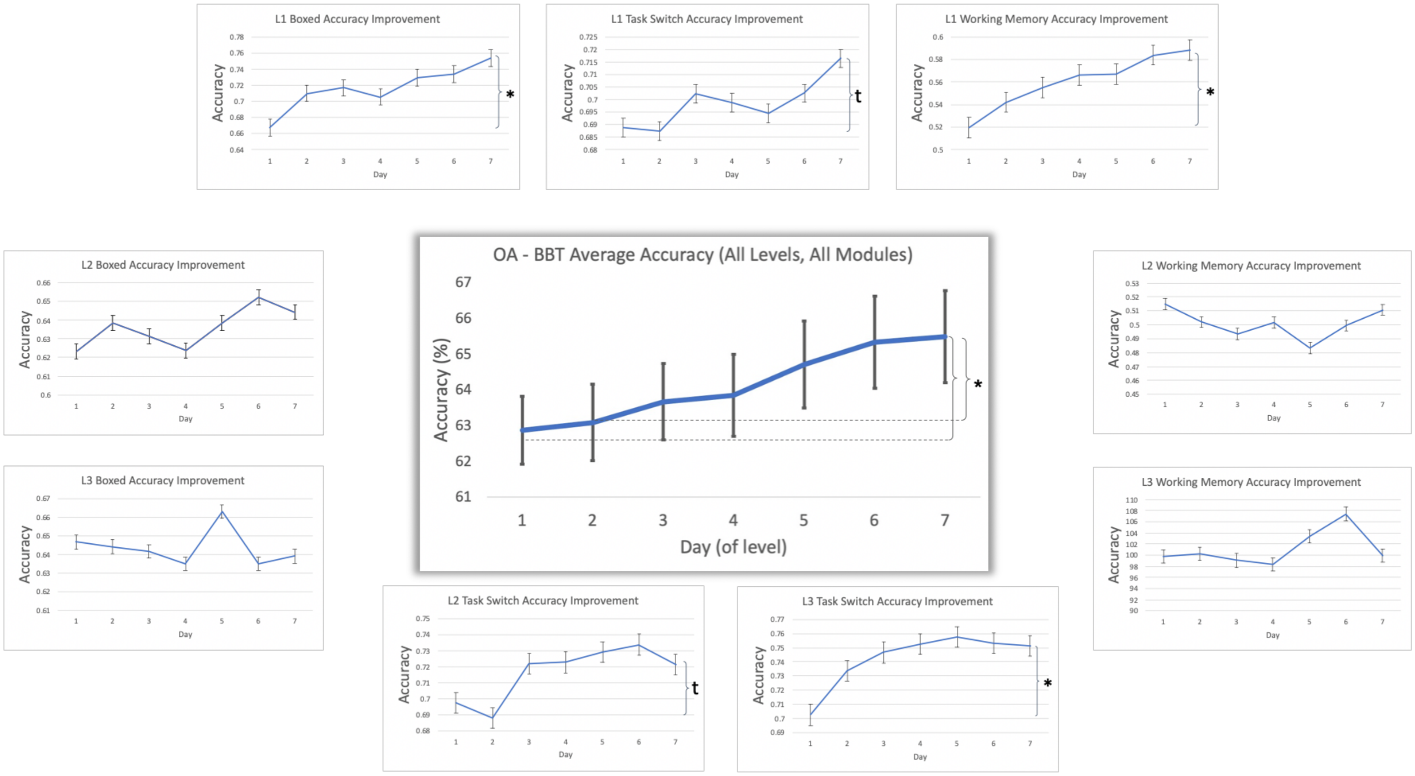

<u>Supplemental Figure 4: Accuracy across BBT training modules over time.</u> During training on BBT, participants significantly increased their accuracy from the start to the end of each module for 3 out of 9 modules. Taking the average of the nine BBT modules (center figure), participants significantly increased their accuracy from the start of training (Days 1 and 2, mean= 63.0%, s.e. = 0.7) to the end (Days 6 and 7, mean= 65.5%, s.e. = 0.7, Δ = 2.5%, paired t-test: t_{22} = -3.583, p = 0.002, 95% CI: -1.204 to -0.277). * = p< 0.05. Error bars represent s.e.m.

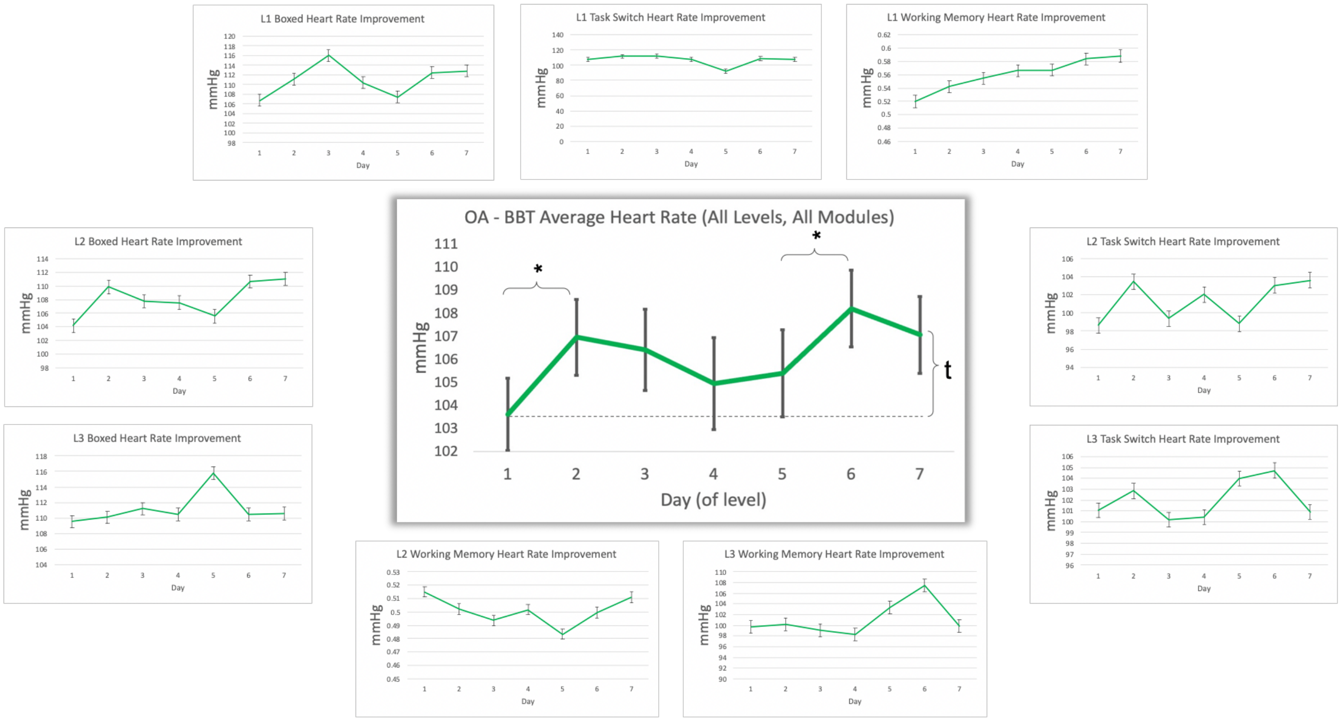

<u>Supplemental Figure 5: Heart Rate across BBT training modules over time</u>: During training on BBT, participants numerically increased their heart rate from the start to the end of each module for 8 out of 9 modules, but none of the changes were significant. Taking the average of the nine BBT modules (center figure), participants showed a trending increase of their heart rate from the start of training (Day 1, mean= 103.6, s.e. = 0.919) to the end (Day 7, mean= 107.1, s.e. = 0.997, Δ = 3.44 mmHG, paired t-test: t_{22} = -1.925, p = 0.056, 95% CI: -0.306 to 0.004). * = p< 0.05 Error bars represent s.e.m.

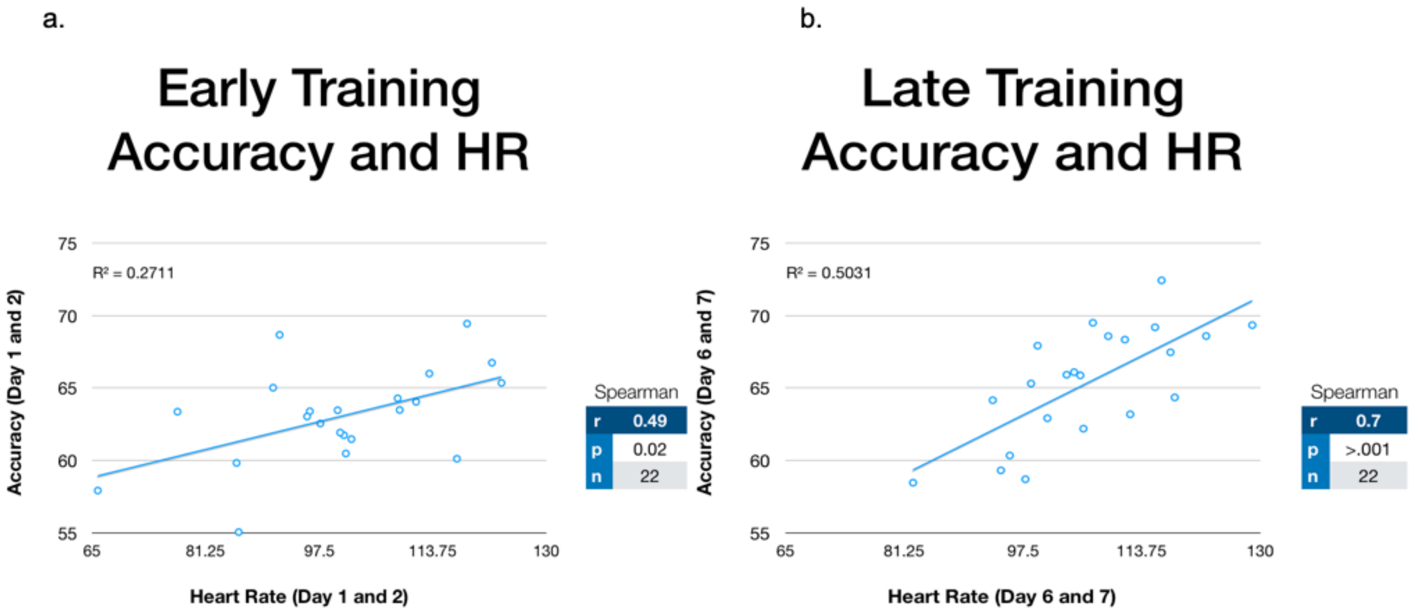

Supplemental Figure 6: Correlation between heart rate and training accuracy. There was a significant correlation between training accuracy and heart rate, such that the participants who had the higher heart rate, gained a higher cognitive benefit during training. This was the case both early in training (The average of Days 1 and 2, Spearman $r_{22} = 0.49$, $P = 0.02$) as well as late in training (The average of Days 6 and 7, Spearman $r_{22} = 0.7$, $P < 0.001$).

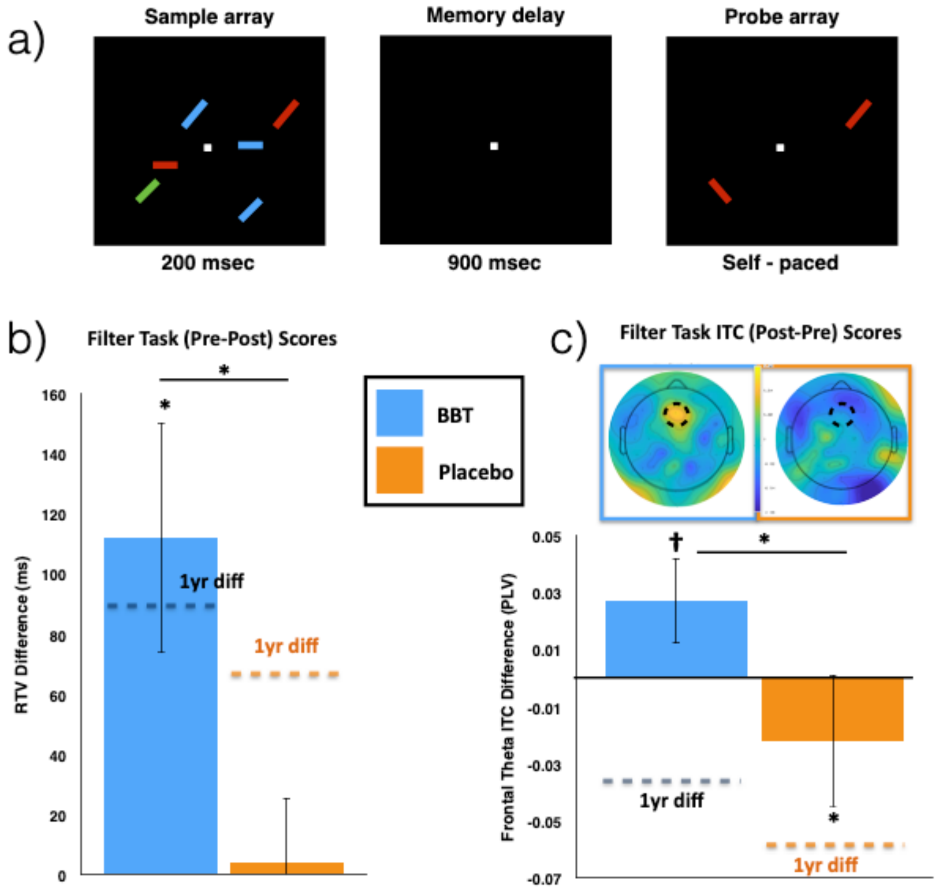

Supplementary Figure 7. Attention with Distraction Task (Filter). a, Stimuli and protocol for the attention with distraction (Filter) task. b, Bar graphs illustrating the group mean change (pre - post) in response time variability (RTV) for each group, with the dashed line (1-year diff = pre - 1-year) illustrating the change in RTV at the 1-year mark. c, Bar graphs and topographic plots illustrating the group mean change (post - pre) in inter-trial coherence (ITC) for each group, with the dashed line on the bar graphs illustrating the change in ITC at the 1-year mark (1-year - pre). The dashed circle on the topographic plot illustrates the electrodes where statistical analyses took place. * = $p < .05$. † = $p = .08$. Error bars represent s.e.m.

__Attention with Distraction (Filter task):__

Given that this task was not a part of the preregistration battery, we performed an exploratory analysis with a more challenging complex visual discrimination task with varying levels of distraction

(**Supplementary Figure 7a**; see **Methods** for task details) to see if comparable improvements (as on the CPT task) might emerge. We chose to evaluate a similar metric to ex-gaussian tau, response time variance (RTV), on this task given that we have previously used this particular measure to assess cognitive enhancement following an intervention[5]. A repeated measures ANOVA evaluating RTV across trials revealed a significant set size (1 or 3 items to be remembered) X time (Pre, Post) X intervention group (BBT, Control) interaction ($F_{(1,42)}$=5.70, p=0.022, Cohen's d = .73). For set size one, follow-up tests revealed performance-related improvements (i.e., less variability) for both the BBT group (Δ = +77.3 msec improvement, paired t-test: t_{24} = 3.26, p = 0.003) and the MBT group (Δ = +61.6 msec improvement, paired t-test: t_{23} = 2.97, p = 0.007). However, for set size three, follow-up tests evidenced performance-related improvements for only the BBT group (Δ = +112.55 msec improvement, paired t-test: t_{20} = 2.93, p = 0.008 for set size of 3), and not for the MBT group (Δ = +4.20 msec improvement, paired t-test: t_{22} = 0.20, p = 0.85 for set size of 3 (**Supplementary Figure 7b**)). With respect to set size three RTV performance one year later, paired t-tests revealed that the BBT group (t_{12} = 1.01, p= .32) showed comparable performance versus their post-training levels. While unexpected, the positive gains by the MBT group at the 1-year mark (paired t-test: t_{12} = 3.25, p= .006) is consistent with previous work demonstrating heightened practice effects in older adults on especially demanding tasks[6-9]. When compared to the group of young adults, the BBT group showed lower performance (greater pre-training RTV) (independent t-test: t_{74} = 2.93, p= .003; see **Supplementary Figure 8**). However, after the intervention period, the BBT group's post-training performance on set size 3 reached an equivalent level of performance (independent t-test: t_{71} = .08, p= .94; **Supplementary Figure 8**).

<u>Neural Correlates of Attention with Distraction:</u>

We also assessed neural correlates associated with training-related improvements in the complex visual discrimination task. Here we examined frontal midline theta inter-trial coherence (ITC), a measurement of trial by trial phase consistency as ITC has been shown to correlate with RTV, the behavioral metric examined on this task[5,10] and is sensitive to intervention-based changes[5,10]. ITC is a measure that reflects the extent to which synchronization occurs from trial to trial in EEG at a particular frequency and latency[11]. In other words, it is a measure of electrophysiological response consistency comparable to the previously described behavioral metrics of ex-gaussian tau and RTV. ITC is quantified by the unit "phase locking value" (PLV), which ranges anywhere between 0 and 1, with a

value of 0 indicating that the phase synchrony is completely random, and a value of 1 indicating that the phase-locking is perfectly synchronized across trials. ITC is defined as: ITC(f,t) = 1n∑k = 1nF_k(f,t)|F_k(f,t)|.

Theta ITC analyses were conducted across all trial types and specifically during the set size of 3 condition of the filter task in accord with the behavioral outcome findings. Based on previous work examining theta ITC during cognitive control tasks[12-18], we focused our analyses on the Fz electrode. All calculated ITC PLVs were controlled for individual state differences at each session by baseline correcting each individual's PLVs using their -200 to 0 period prior to stimulus onset (thus, a relative PLV score was calculated for each subject), as in our previous work[4,19]. To examine a particular time window of interest, we first segmented the resolved ITC time series into 100 msec bins following stimulus onset. To select a particular time window of interest for interrogation, we first conducted a repeated measures ANOVA with session (pre and post) and time window (0-600 in 100msec bins) as the within subjects factors, and group (BBT and MBT) as the between subjects factor. The session by time window by group interaction approached significance ($F_{(4,120)}$= 2.20, p=0.09), with the session by group interaction showing a significant effect ($F_{(1,30)}$= 4.83, p=0.036) supporting a differential improvement in ITC for the BBT group beyond that of the MBT group over all time windows. Given the strong trend for the session by time window by group interaction and our desire to avoid collapsing across all time windows at the individual subject level for correlational analyses, we chose to conduct follow-up repeated measure ANOVAs at each time window (0-100, 100-200, etc) with time (pre and post) as the within subjects factor and group (BBT or MBT) as the between subjects factor. The p-values from each group x time interaction underwent an FDR correction for multiple comparisons, and the time window nearest to stimulus onset that survived the FDR correction (300-400 msec; corrected p = 0.016) was used to select our time window of interest here.

Given that the behavioral results revealed that the BBT group selectively improved beyond the MBT group on the more difficult conditions of this task (set size 3), we focused the neural analyses during this condition. Here, a repeated measures ANOVA with a within-subject factor of time (Pre, Post) and a between-subject factor of Study Group (BBT, Control) showed a significant Group by Time interaction ($F_{(1,39}$=7.98, p=0.007, Cohen's d = .91; **Supplementary Figure 7c**). Post-hoc paired t-tests revealed an improvement that was trending toward significance (i.e., higher ITC) in the BBT group (Δ = 0.027, paired t_{20} = 1.87, p = 0.08), and a significant decrease in the MBT group (Δ = -0.022, paired t_{19} =

-2.40, p = 0.03). With respect to maintenance of any neural changes 1 year later, follow-up paired tests revealed a both groups regressed from their post-training state (BBT: t_{13}= 2.15, p= .05; MBT: t_{13}= -2.89, p= .01; see **Table 1** for values). When compared to young adults via independent t-tests, both BBT and MBT post-training ITC did not differ to young adults at baseline (BBT: t_{54} = 50, p= .62; MBT: t_{53} = 1.00, p= .34) or after the intervention period (BBT: t_{48} = .65, p= .52; MBT: t_{51} = .74, p= .46; **Supplementary Figure 8**).

Supplementary Figure 8. Comparison of BBT and MBT performance versus younger adults.

	BBT Pre-Training	MBT Pre-Training	BBT Post-Training	MBT Post-Training
**Filter (RTV)**	⬇	=	= (green)	=
**Filter (mfTheta ITC)**	=	=	=	=

⬇ => performance significantly lesser than that of young adults

⬆ => performance significantly better than that of young adults

= => performance equivalent to that of young adults

Green = Significant improvement from pre-training

<u>Sustained Attention (CPT) RT and RTV results</u>

For response time, using the same repeated measures ANOVA approach as in the main manuscript to evaluate the change in performance from pre-intervention to post-intervention, we found no significant time (Pre, Post) by intervention group (BBT, MBT) interaction ($F_{1,42}$= 1.91, p = 0.17), nor a main effect of session ($F_{1,42}$= 0.86, p = 0.771). Using the same statistical approach, a similar pattern of effects was observed for response time variance with respect to the interrogated interaction ($F_{1,42}$= 1.23, p = 0.27) and the main effect of session ($F_{1,42}$= 0.59, p = 0.44).

Physical outcome measures:

Here we describe other exploratory measures collected and assessed beyond the two primary measures of diastolic blood pressure and limits of stability. When we evaluated systolic blood pressure, we observed the BBT group showed a trend towards improvement following training (paired t-test: t_{21}= 1.94, p= 0.065), unlike the MBT group (paired t-test: t_{23} = -0.27, p= 0.79). However, the repeated measures ANOVA analysis did not evidence a significant group by time interaction ($F_{(1,45)}$= 2.28, p= 0.13), and at baseline, the BBT group showed a trend towards having greater systolic blood pressure than the MBT group (independent t-test: t_{45}= 1.78, p= .08). For systolic blood pressure, the ANCOVA approach replicated the previously described null interaction effect ($F_{(1,44}$= .40, p= 0.53).

As exploratory measures, we performed physical assessments from the Senior Fitness Test (SFT) to assess global physical performance[20]. Assessments from the SFT battery included: Chair Stand (lower body strength), Arm Curl (upper body strength), Chair Sit-and-Reach Left and Right (lower body flexibility), Back Scratch Left and Right (upper body flexibility), the (8-foot) Timed Up and Go (agility), and Gait Speed (preferred and fast). Note that none of these exploratory measures showed a group X time interaction (Chair Stands: $F_{(1,45)}$= 2.5, p= .12, Arm Curls: $F_{(1,45)}$= 1.72, p= .20, Chair Sit-and-Reach Left: $F_{(1,45)}$= 1.18, p= .28, Chair Sit-and-Reach Right: $F_{(1,45)}$= 0.34, p= .56, Back Scratch Left: $F_{(1,45)}$= 0.07, p= .80, Back Scratch Right: $F_{(1,45)}$= 0.03, p= .86, Timed Up and Go: $F_{(1,45)}$= 0.37, p= .55, Gait Speed preferred: $F_{(1,45)}$= 0.056, p= .82, Gait Speed fast: $F_{(1,45)}$= 1.32, p= .26).

It should be noted that we asked all participants to not change any aspects of their daily routine throughout the duration of the study. Nevertheless, we also collected information from each group at the 1-year mark using the Physical Activity Scale for the Elderly[21,22] (PASE) survey to assess if there were differences in physical activities undertaken after the intervention. Analyses of the PASE revealed no difference between the groups at the 1-year mark (independent t-test: $t_{(42)}$= .87, p= .26).

References

1 Boot, W. R., Simons, D. J., Stothart, C. & Stutts, C. The pervasive problem with placebos in psychology why active control groups are not sufficient to rule out placebo effects. . *Perspect. Psychol. Sci.* **8**, 445–454 (2013).

2 Simons, D. J. *et al.* Do "Brain-Training" Programs Work? *Psychol Sci Public Interest* **17**, 103-186, doi:10.1177/1529100616661983 (2016).

3 Mishra, J., Anguera, J. A. & Gazzaley, A. Video Games for Neuro-Cognitive Optimization. *Neuron* **90**, 214-218, doi:10.1016/j.neuron.2016.04.010 (2016).

4 Anguera, J. A. *et al.* Video game training enhances cognitive control in older adults. *Nature* **501**, 97-101, doi:10.1038/nature12486 (2013).

5 Ziegler, D. A. *et al.* Closed-loop digital meditation improves sustained attention in young adults. *Nat Hum Behav* **3**, 746-757, doi:10.1038/s41562-019-0611-9 (2019).

6 Wais, P. E. & Gazzaley, A. External distraction impairs categorization performance in older adults. *Psychol Aging* **29**, 666-671, doi:10.1037/a0037617 (2014).

7 McCarley, J. S., Yamani, Y., Kramer, A. F. & Mounts, J. R. Age, clutter, and competitive selection. *Psychol Aging* **27**, 616-626, doi:10.1037/a0026705 (2012).

8 Becic, E., Boot, W. R. & Kramer, A. F. Training older adults to search more effectively: scanning strategy and visual search in dynamic displays. *Psychol Aging* **23**, 461-466, doi:10.1037/0882-7974.23.2.461 (2008).

9 Rolle, C. E., Anguera, J. A., Skinner, S. N., Voytek, B. & Gazzaley, A. Enhancing Spatial Attention and Working Memory in Younger and Older Adults. *Journal of Cognitive Neuroscience* **29**, 1483-1497, doi:10.1162/jocn_a_01159 (2017).

10 Lutz, A. *et al.* Mental training enhances attentional stability: neural and behavioral evidence. *J Neurosci* **29**, 13418-13427, doi:10.1523/JNEUROSCI.1614-09.2009 (2009).

11 Tallon-Baudry, C., Bertrand, O., Delpuech, C. & Pernier, J. Stimulus specificity of phase-locked and non-phase-locked 40 Hz visual responses in human. *J Neurosci.* **16**, 4240-4249. (1996).

12 Bishop, D. V. & Hardiman, M. J. Measurement of mismatch negativity in individuals: a study using single-trial analysis. *Psychophysiology* **47**, 697-705, doi:10.1111/j.1469-8986.2009.00970.x (2010).

13 Busch, N. A., Dubois, J. & VanRullen, R. The phase of ongoing EEG oscillations predicts visual perception. *J Neurosci* **29**, 7869-7876, doi:10.1523/JNEUROSCI.0113-09.2009 (2009).

14 Caravaglios, G., Castro, G., Muscoso, E. G., Crivelli, D. & Balconi, M. Beta Responses in Healthy Elderly and in Patients With Amnestic Mild Cognitive Impairment During a Task of Temporal Orientation of Attention. *Clin EEG Neurosci* **49**, 258-271, doi:10.1177/1550059416676144 (2018).

15 Ko, D. *et al.* Theta oscillation related to the auditory discrimination process in mismatch negativity: oddball versus control paradigm. *J Clin Neurol* **8**, 35-42, doi:10.3988/jcn.2012.8.1.35 (2012).

16 Muller, V. & Anokhin, A. P. Neural synchrony during response production and inhibition. *PLoS One* **7**, e38931, doi:10.1371/journal.pone.0038931 (2012).

17 Wang, W., Viswanathan, S., Lee, T. & Grafton, S. T. Coupling between Theta Oscillations and Cognitive Control Network during Cross-Modal Visual and Auditory Attention: Supramodal vs Modality-Specific Mechanisms. *PLoS One* **11**, e0158465, doi:10.1371/journal.pone.0158465 (2016).
18 Lutz, A. *et al.* Mental training enhances attentional stability: neural and behavioral evidence. *The Journal of neuroscience : the official journal of the Society for Neuroscience* **29**, 13418-13427 (2009).
19 Anguera, J. A. *et al.* Long-term maintenance of multitasking abilities following video game training in older adults. *Neurobiol Aging* **103**, 22-30, doi:10.1016/j.neurobiolaging.2021.02.023 (2021).
20 Jones, C. J. & Rikli, R. E. Measuring functional fitness of older adults. *The Journal on active aging* **1**, 24-30 (2002).
21 Washburn, R. A., McAuley, E., Katula, J., Mihalko, S. L. & Boileau, R. A. The physical activity scale for the elderly (PASE): evidence for validity. *J Clin Epidemiol* **52**, 643-651, doi:10.1016/s0895-4356(99)00049-9 (1999).
22 Washburn, R. A., Smith, K. W., Jette, A. M. & Janney, C. A. The Physical Activity Scale for the Elderly (PASE): development and evaluation. *J Clin Epidemiol* **46**, 153-162, doi:10.1016/0895-4356(93)90053-4 (1993).
